# The impossible challenge of estimating non-existent moments of the Chemical Master Equation

**DOI:** 10.1093/bioinformatics/btad205

**Published:** 2023-06-30

**Authors:** Vincent Wagner, Nicole Radde

**Affiliations:** Institute for Systems Theory and Automatic Control, University of Stuttgart, Stuttgart 70569, Germany; Institute for Systems Theory and Automatic Control, University of Stuttgart, Stuttgart 70569, Germany; Stuttgart Center for Simulation Science, University of Stuttgart, Stuttgart 70569, Germany

## Abstract

**Motivation:**

The Chemical Master Equation (CME) is a set of linear differential equations that describes the evolution of the probability distribution on all possible configurations of a (bio-)chemical reaction system. Since the number of configurations and therefore the dimension of the CME rapidly increases with the number of molecules, its applicability is restricted to small systems. A widely applied remedy for this challenge is moment-based approaches which consider the evolution of the first few moments of the distribution as summary statistics for the complete distribution. Here, we investigate the performance of two moment-estimation methods for reaction systems whose equilibrium distributions encounter fat-tailedness and do not possess statistical moments.

**Results:**

We show that estimation via stochastic simulation algorithm (SSA) trajectories lose consistency over time and estimated moment values span a wide range of values even for large sample sizes. In comparison, the method of moments returns smooth moment estimates but is not able to indicate the non-existence of the allegedly predicted moments. We furthermore analyze the negative effect of a CME solution’s fat-tailedness on SSA run times and explain inherent difficulties. While moment-estimation techniques are a commonly applied tool in the simulation of (bio-)chemical reaction networks, we conclude that they should be used with care, as neither the system definition nor the moment-estimation techniques themselves reliably indicate the potential fat-tailedness of the CME’s solution.

## 1 Introduction

Randomness and uncertainty govern nearly every aspect of our life. This especially also holds for the evolution of (bio-)chemical reaction networks (CRNs). As the quality and quantity of biochemical data have increased dramatically over the last decades, modeling a process’s noise has simultaneously become a valuable part of research (Tsimring 2014). According to Mandelbrot (1997), randomness can be classified into three broad categories: mild, slow, and wild. The prototypical representative of mild randomness is normally distributed noise. Numerous biological processes are assumed to be normally distributed in nature (Tsimring 2014) and this belief can often be motivated by independent additive errors and the central limit theorem. Other biological examples for mild stochasticity arise from simple birth–death processes that can be shown to behave according to Poisson distributions (Wilkinson 2011), and Weidemann et al. (2023) demonstrate that housekeeping genes exhibit an even smaller variance in their gene expression levels.

However, recent results indicate that biochemical stochasticity is often significantly less well-behaved than previously assumed. Beal (2017), e.g. argues that gene expression without other dominant factors is best described by log-normal distributions and later findings consolidate this result (Ham et al. 2020; Jia and Grima 2020; Bokes 2021). Log-normal errors arise from positive, multiplicative errors and this kind of stochasticity falls into the second class of slow randomness. Lastly, wild randomness is characterized by single realizations that dominate the overall process completely.

In biological settings, but also in nearly every other stochastic context, a very popular coping strategy for randomness is based on the formulation of expectations. Generally, an expectation is a deterministic way of describing a stochastic process by calculating the average of several of its outcomes. Mathematically, the notion of expectation manifests in the form of statistical moments like the expectation value and the variance. These quantities, as well as their estimation, are well-understood. Furthermore, their use in describing stochastic processes is provably founded by the central limit theorem: For processes of finite expectation and variance, the theorem states that the average over several independent realizations converges to a normally distributed random variable with a shrinking variance for a growing number of averaged samples. While the central limit theorem is remarkable in every aspect, its implications are still limited to processes that are expectable in the notion of statistical moments.

**Figure 1.**
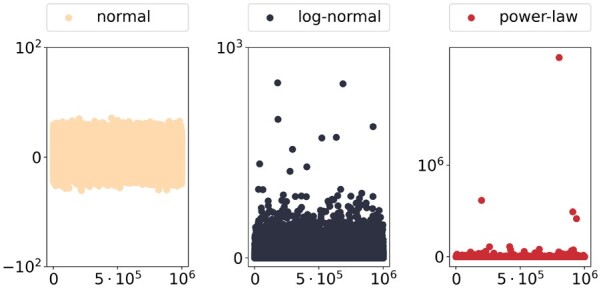
Mild, slow, and wild randomness exemplified by samples from three distributions originating from (bio-)chemical reaction systems. Each of the three plots presents an independent and identically distributed sample of size $10^{6}$ for a different distribution. On the left side, a normal random variable is sampled, the center shows a sample from a log-normal distribution and the sample on the right originates from a distribution with probability measure $P(X=k)\propto \frac{1}{k^{2}}$. The distributions corresponding to the left and central sample have the same expectation value E=e and variance $V=e^{4}-e^{2}$, while the last distribution does not possess any statistical moments.

Figure 1 illustrates the three discussed types of randomness. While the impact of single outliers is basically non-existent for the normal random variable, this changes for the log-normal case, even though both distributions have the same expectation value and variance. The sample of the inverse quadratic distribution is dominated by four realizations and therefore clearly wildly stochastic. This wild stochasticity results in non-existent moments.

Wildly stochastic behavior has been described, i.e. in financial sciences, where 63% of financial returns of half a century can be associated with only 10 trading days (Mandelbrot and Taleb 2006). So far, it remains unclear whether such phenomena also appear in biological systems. We show here that wild stochasticity can be obtained by simple mass action systems with quadratic propensities.

Before constructing a wildly stochastic CRN, we first need a modeling framework to describe its general behavior. The Chemical Master Equation (CME) is a standard approach for this purpose (see e.g. Higham 2008). Its solution is a time-dependent probability distribution over all configurations of the system. Statistical moments of this distribution can be estimated either via Monte Carlo integration over sample paths from the underlying process, which can be generated via the stochastic simulation algorithm (SSA; Gillespie 1976, 1977), or by the method of moments (MoM; Engblom 2006), a differential equation system for the moments that can be solved via numerical integration. On this basis, more sophisticated methods can be created. Some combine SSA sampling and the MoM using extended Kalman fitlering (Ruess et al. 2011), others choose either of the methods dynamically depending on the size of the simulated chemical populations (Henzinger et al. 2010; Hasenauer et al. 2014). Henzinger and Mateescu (2011) complement the estimation of statistical moments with approximations of the distribution’s tail and Kuntz et al. (2019) bound the moments of the stationary distribution of the CME using mathematical programming. We do not know how these methods behave for CME solutions without existing moments. Beyond the mentioned approaches, Prugger et al. (2022) and Fang et al. (2022) subdivide the overall CRN into different biologically meaningful subsets. The resulting set of smaller systems can then be solved easier and faster than the overall network. Prugger et al. (2022) exchange averaged information between the subsets but do not make this simplification within each subset. Fang et al. (2022), on the other hand, simulate several SSA traces for the main sub-system and solve the CME directly for all other sub-systems that are conditioned on the main one. In a fundamentally different approach, Sukys et al. (2022) approximate the solution of the CME using surprisingly simple neural networks. The networks are trained by few SSA samples and therefore potentially circumvent the troubles of non-existent moments.

We will nevertheless focus on the two fundamental approaches since most other approaches pose additional conditions to the CRN and are therefore not necessarily better suited to answer a scientific question at hand. In this work, SSA sampling and the MoM are applied to a CRN that converges to a power-law distribution without existing moments like the one presented in Fig. 1 and consequently shows wildly stochastic behavior. The results are compared with results originating from a very well-behaved network. We demonstrate that expectation estimation techniques for CRNs are not capable of indicating wild stochasticity but instead return meaningless results, thereby deceiving inattentive users.

## 2 Systems and methods

### 2.1 Expectation-based approximation techniques for the CME solution

We consider a CRN with *N* molecule types that participate in *J* reactions. The configuration space of such a system is a set of system configurations described by a vector $\vec{x}\in R^{N}$ whose entries $x_{i}\in N_{0}$ correspond to the number of molecules of species *i*. A reaction *j* is assigned a configuration change vector ${\vec{\nu }}_{j}$, which comprises the changes in molecule numbers if reaction *j* fires once, and a propensity function $a_{j}(\vec{x})$. The product $a_{j}(\vec{x})dt$ indicates how likely reaction *j* fires in an infinitesimal time instance [t,t+dt). This defines the CME, whose solution is a time dependent probability distribution $P(\vec{x},t)$ on the space of system configurations. The propensity $a_{j}(\vec{x})$ can be chosen by using the law of mass action, according to which the probability of a reaction is proportional to the number of possible educt combinations. The CME describes a time-continuous Markov process on the discrete space of configurations. These processes often converge to an equilibrium distribution, which means that the limit exists independent of the initial condition. For clarity and brevity of notation, we restrict our considerations to one-dimensional configuration spaces, i.e. we consider CRNs with one molecular species only. Results can in principle directly be generalized, but the numbers of expectation values and covariances increase linearly and quadratically with the number of molecular species, respectively.


(1) $$\frac{dP(\vec{x},t)}{dt}=\sum_{j=1}^{J}a(\vec{x}-{\vec{\nu }}_{j})P(\vec{x}-{\vec{\nu }}_{j},t)-a(\vec{x})P(\vec{x},t),$$

(2) $$\bar{P(\vec{x})}=\underset{t\to \infty }{\lim}P(\vec{x},t)$$

We are particularly interested in the time-dependent first two moments of the probability distribution, the expectation value, and the variance, which are defined via


(3) $$\begin{array}{c} E_{x}(t)=\underset{K\to \infty }{\lim}\sum_{k=0}^{K}P(x=k,t)\;k \end{array}$$

(4) $$V_{x}(t)=\underset{K\to \infty }{\lim}\sum_{k=0}^{K}P(x=k,t){\left(k-E_{x}(t)\right)}^{2}.$$

The somewhat lengthy limit formulation stresses that this indeed is a series for which convergence is not trivially given. Throughout this article, we refer to distributions without converging moment series as fat-tail distributions. This term is ambiguous and our definition is one of the more restrictive ones. We especially would like to distinguish this definition from heavy-tailed distributions, as many researchers use this term also for the log-normal case that we associated with slow randomness in the introduction. Two complementary approaches approximate $E_{x}(t)$ and $V_{x}(t)$. First, moments are estimated via simulating sample trajectories from the CME, which is realized via the SSA and Monte Carlo integration of these trajectories for moment estimation. The SSA is easy to implement and can readily be applied for many systems of interest. However, especially for large systems including many molecules, each single SSA trajectory is expensive to compute and many samples are needed to reach a sufficiently converged moment estimator, as its convergence is slow. When using too few samples, the estimated moment trajectories are rather unsteady.

As a second approach, we use the MoM, which derives a set of differential equations for the first *k* moments from the CME. These equations are not closed for systems with reactions of order two or higher, since lower-order moments depend on higher-order moments in these cases. We use truncation closure (TC) to solve this problem, a closure method that neglects the influences of higher-order moments on the considered set of moment equations. Unsurprisingly, it is, therefore, one of the most simplistic approaches, and we refer the interested reader to more sophisticated closure techniques that have been proposed to break this infinite hierarchy (see e.g. Ruess et al. 2011; Grima 2012; Wagner et al. 2022). There is no general result that characterizes the optimal number of included moments or the accuracy of moment equations. However, the MoM results are typically smoother and faster to calculate compared with SSA-based estimations and therefore often favorable in practical applications. As an example, consider MoM approximations for the expectation value $E_{x}(t)$ and variance $V_{x}(t)$ of the number of molecules *x* in a chemical system with only one species. After applying TC and thereby neglecting the influence of the skewness on the first two moments, the Taylor series expansion of the MoM equations reads


(5) $$\begin{array}{cc} \frac{d{\tilde{E}}_{x}(t)}{dt} & =\sum_{j=1}^{J}\nu_{j}\left(a_{j}\left({\tilde{E}}_{x}(t)\right)+\frac{d^{2}a_{j}\left({\tilde{E}}_{x}(t)\right)}{dx^{2}}\frac{{\tilde{V}}_{x}(t)}{2!}\right) \end{array}$$

(6) $$\begin{array}{c} \frac{d{\tilde{V}}_{x}(t)}{dt}=\sum_{j=1}^{J}2\nu_{j}\left(\frac{da_{j}\left({\tilde{E}}_{x}(t)\right)}{dx}\frac{{\tilde{V}}_{x}(t)}{1!}\right)\\ +\sum_{j=1}^{J}\nu_{j}^{2}\left(a_{j}\left({\tilde{E}}_{x}(t)\right)+\frac{d^{2}a_{j}\left({\tilde{E}}_{x}(t)\right)}{dx^{2}}\frac{{\tilde{V}}_{x}(t)}{2!}\right). \end{array}$$

Here, the tilde denotes the ordinary differential equation (ODE)-based approximation of the underlying quantity due to TC.

### 2.2 Deriving a chemical reaction system that provably exhibits wildly stochastic behavior

In order to study the behavior of both moment-estimation methods for systems that exhibit wildly stochastic behavior, we inversely design such a system. To this end, we consider the steady-state condition of the CME, which is obtained by setting the time derivative in Equation (1) to zero and reads


(7) $$\sum_{j=1}^{J}a_{j}(x-\nu_{j})\bar{P(x-\nu_{j})}-\sum_{j=1}^{J}a_{j}(x)\bar{P(x)}=0.$$

Reformulating our original goal, we would like to define a CRN whose equilibrium distribution $\bar{P(x)}$ does not possess any statistical moments. One example of such a distribution on the natural numbers is given by


(8) $$P(x=k)=\frac{6}{{\left(\pi k\right)}^{2}},\;k\in N.$$

Equation (8) defines a proper, discrete, normalized probability measure on all natural numbers, as


(9) $$\underset{K\to \infty }{\lim}\sum_{k=1}^{K}P(x=k)=1.$$

However, all moments of natural order *m* are defined by divergent sums,


(10) $$\underset{K\to \infty }{\lim}\sum_{k=1}^{K}P(x=k)\;k^{m}\to \infty .$$

This holds regardless of whether one is interested in centralized moments or non-centralized counterparts.

The distribution defined in Equation (8) indeed describes the equilibrium distribution of a birth–death process with quadratic propensities. To illustrate this, we define the CRN


(11) $$\begin{array}{c} 2A\to A|\nu_{1}=-1|a_{1}(x)=c_{1}[x\geq 2]x^{2}\\ 2A\to 3A|\nu_{2}=1|a_{2}(x)=c_{2}x^{2}. \end{array}$$

Here, [⋅] is a Boolean expression that is either equal to zero or one depending on the truth of the argument. Inserting our specific choice of system characteristics into Equation (7) and setting the reaction rate constants $c_{1}=c_{2}=1$ results in


(12) $${\left(x+1\right)}^{2}\bar{P(x+1)}+{\left(x-1\right)}^{2}\bar{P(x-1)}-2x^{2}\bar{P(x)}=0.$$

By replacing all three instances of $\bar{P}$ in Equation (12) using Equation (8), we obtain which holds for all *x*.


(13) $${\left(x+1\right)}^{2}\frac{6}{\pi^{2}{\left(x+1\right)}^{2}}+{\left(x-1\right)}^{2}\frac{6}{\pi^{2}{\left(x-1\right)}^{2}}-2x^{2}\frac{6}{\pi^{2}x^{2}}=0,$$

In summary, we defined a CRN whose equilibrium CME solution is a discrete fat-tail distribution: it neither possesses an expectation value nor any higher-order moment.

**Figure 2.**
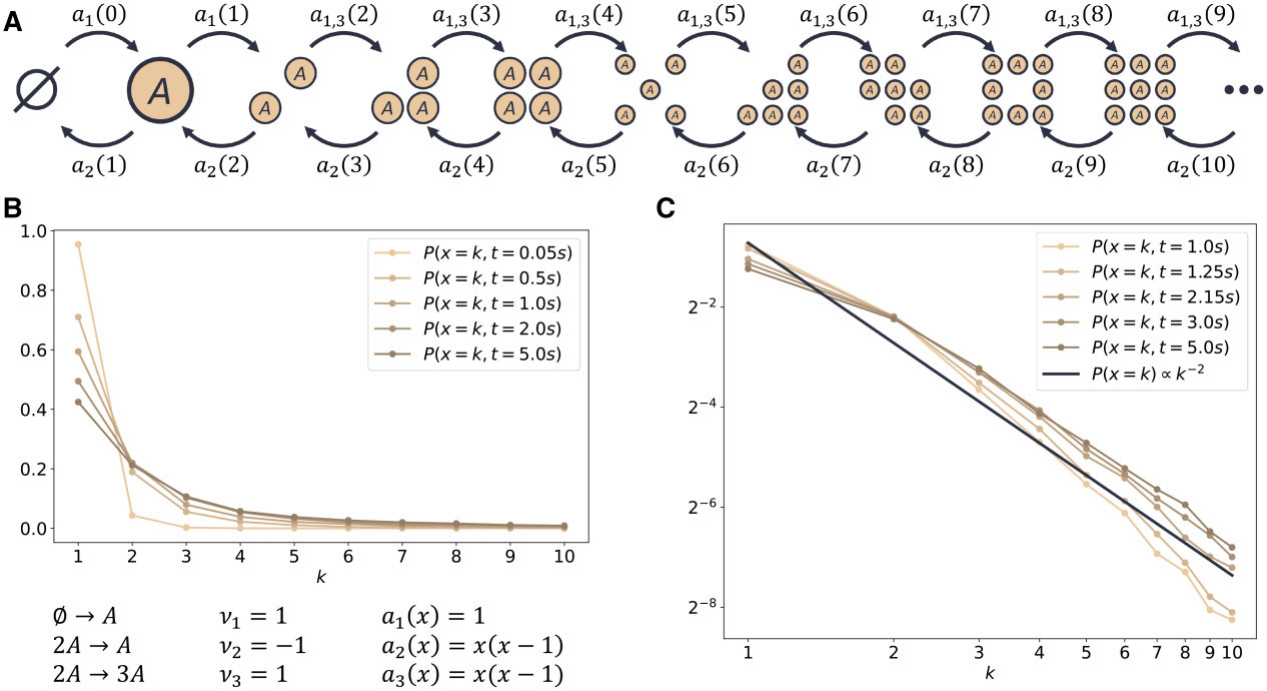
A birth–death process that exhibits wildly stochastic behavior. (A) Configuration transition graph, (B) P(x,t) estimated with 25 000 SSA trajectories that are evaluated over a logarithmically scaled, uniform time grid with 1001 time points, P(x,t=0) was set to P(x=1,0)=1. (C) A polynomial decay of distribution tails corresponds to a straight line in the double-logarithmic plot, as indicated with the black line of the fat-tail distribution (8). The distribution P(x,t) also approaches such a straight line for later time points and k≤2, thus indicating fat-tailedness.

Our system is unphysical in two aspects. First, defining the propensities for second-order reactions according to the law of mass action, one counts the number of educt combinations, which is given by x(x−1) instead of $x^{2}$. This detail matters in particular for small molecule numbers. The second unphysical aspect is the Boolean expression that prohibits an absorbing system conformation at x=0. In order to avoid this, we introduce a third reaction that slowly but constantly adds species *A* to the system. The resulting configuration transition graph of this modified system, which we refer to as System W, is shown in Fig. 2A. It is more involved to derive the steady-state distribution for this altered system, which is why we resort to numerical simulations to validate our claims for realistic scenarios further. We nevertheless expect System W to have very similar characteristics because of nearly identical propensities, especially in the realm of many molecules. The time evolution of the system that is obtained from 25 000 SSA sample trajectories with initial configuration x(0)=1 is illustrated in Fig. 2B for five exemplary time points. As expected, a shift of the probability mass toward larger numbers of molecules can be observed.

An obvious question at this point is whether System W indeed possesses a fat-tail steady-state solution. Figure 2C displays the evolution of the CME solution approximation of System W in comparison to the fat-tail distribution (8). Since both axes are logarithmically scaled, the polynomial distribution (8) is a straight line with a defined slope. Distributions that decay faster than this reference correspond to lines with steeper slopes and vice versa. One clearly sees that the slopes of the CME solutions decay with growing time, eventually intersecting the slope of distribution (8). This point specifically marks the change from a CME solution with defined moments to a fat-tailed one without. Therefore, the CME solution P(x,t) of System W indeed makes a transition from an initial distribution with defined moments for early time instances to a fat-tail distribution for later instances, and hence can be used for the analysis of moment-based approaches.

**Figure 3.**
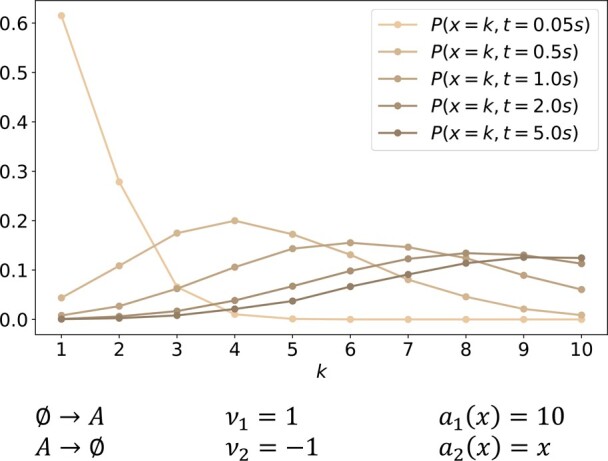
A simple immigration-death process with first-order kinetics serves as a benchmark problem. This system converges to a Po(10) distribution with well-defined moments. P(x,t=0) has been set to P(x=1,0)=1.

To put our findings into context, all simulations are similarly performed for a reference System P with well-defined moments depicted in Fig. 3. This system is a simple immigration-death process with first-order kinetics for both reactions. The configuration transition graph can be adopted from System W by neglecting the third reaction (which corresponds to setting $a_{3}(x)=0$). System P converges to a Poisson distribution with parameter λ=10, i.e. the equilibrium distribution is $\bar{P(x)}∼Po(10)$, with expectation and variance $E_{x}=V_{x}=10$.

### 2.3 Moment estimators from SSA trajectories do not converge if the system exhibits wildly stochastic behavior

**Figure 4.**
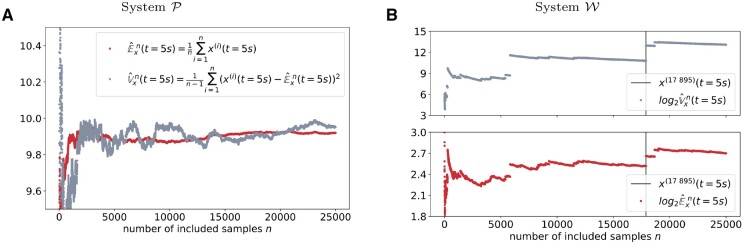
Moment estimators show qualitatively different behaviors for Systems P and W for a growing number of included sample trajectories. Bias-free moment estimators are applied to the final configuration of all sample trajectories x(t=5s) for a growing number of included samples. Red and gray dots correspond to expectation and variance estimations, respectively. (A) Results for System P on a linear scale. (B) Results for System W on a logarithmic scale. Trajectory 17 895 takes the value 10 554 at t=5s.

From the sampled trajectories of the CRN, one can of course still attempt to estimate the distributions’ statistical moments using the bias-free estimators for the expectation values $E_{x}(t)$ and variances $V_{x}(t)$. In this context, index *i* denotes one of the *n* independent SSA trajectories over which we average. Figure 4 compares these moment estimators for Systems P and W over an increasing number of sample trajectories for a time instance t=5s. According to our analysis in Fig. 2C, System W already exhibits wildly stochastic behavior at this point. Following the central limit theorem, one would expect ${\hat{E}}_{x}^{n}$ and ${\hat{V}}_{x}^{n}$ to converge for a growing number of included samples *n* in most scenarios. While this expectation is met for System P (Fig. 4A), the same estimators applied to System W exhibit strikingly different behavior (Fig. 4B): Since subsequent dots differ in exactly one additional sample taken into account during moment estimation, large gaps between subsequent points indicate disruptive trajectories that significantly influence the estimation of the whole trajectory ensemble and thereby prevent convergence. The most disruptive trajectory $x^{\left(17\;895\right)}$ of System W is visually highlighted and marks a huge gap for both moment estimators. Note that the logarithms of both moment estimators have been visualized in order to cope with the large ranges these estimators cover.


(14) $$\begin{array}{c} {\hat{E}}_{x}^{n}(t)=\frac{\sum_{i=1}^{n}x^{\left(i\right)}(t)}{n} \end{array}$$

(15) $${\hat{V}}_{x}^{n}(t)=\frac{\sum_{i=1}^{n}(x^{\left(i\right)}(t)-{\hat{E}}_{x}^{n}(t){)}^{2}}{n-1}$$

Summarizing, Fig. 4 shows that statistical moment estimators do not converge for increasing numbers of SSA trajectories for System W and cover a wide range of values. Therefore, the estimation of moments from a fixed number of trajectories does not lead to reliable estimates.

A reviewer brought another interesting scenario to our attention. By choosing $c_{1}=0$ and $c_{2}=1$, System (11) is effectively reduced to a single growth reaction. Using TC to neglect the variance ${\tilde{V}}_{x}$, the MoM equation (5) for the expectation value reads


(16) $$\frac{d{\tilde{E}}_{x}(t)}{dt}=a_{j}\left({\tilde{E}}_{x}(t)\right)={\tilde{E}}_{x}^{2}(t)$$

For ${\tilde{E}}_{x}(0)>0$, the ODE’s solution is known to diverge in finite time,


(17) $$\underset{t\to {\frac{1}{{\tilde{E}}_{x}(0)}}^{-}}{\lim}{\tilde{E}}_{x}(t)\to \infty .$$

**Figure 5.**
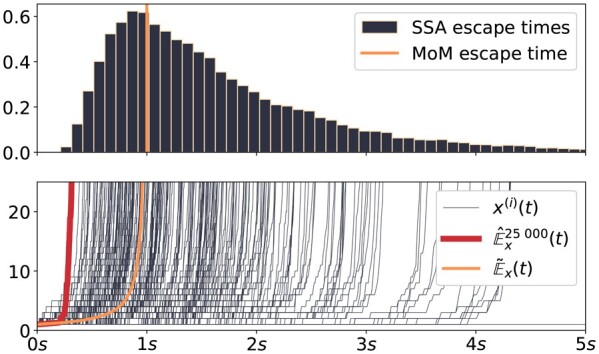
The sample-based expectation estimation and respective MoM prediction for a system with finite escape time do not agree. Comparison of time-dependent moment estimation for a system with finite escape time. 25 000 SSA trajectories were simulated for the system defined in Equation (11) for the case $c_{1}=0,\;c_{2}=1$ and initial condition ${\tilde{E}}_{x}(0)=1$. Since each trajectory eventually escapes in finite time, we set a limit of 10 000 reactions for each of the simulations to finish the simulation runs in finite time. Top: Empirical distribution of escape times estimated from 25 000 SSA runs. Bottom: SSA trajectories $x^{\left(i\right)}$ (black trajectories), expectation value ${\tilde{E}}_{x}(t)$ estimated by the MoM (orange line) and ${\hat{E}}_{x}^{25\;000}(t)$ from SSA trajectories (red line).

Figure 5 illustrates SSA-based and MoM moment-estimation results for this system. The MoM solution ${\tilde{E}}_{x}(t)$ exists for t<1 in this setting (orange line). All SSA trajectories escape in finite but heterogeneous times, as can be seen by the SSA trajectories (black lines bottom) and the empirical escape time distribution (top). Moreover, the SSA-based moment trajectory (red line) also diverges, but much faster than ${\tilde{E}}_{x}(t)$, which indicates that the sample average is at time points t<1 already dominated by extreme events. On this basis, we reason that similar to system W, this finite escape-time system does not possess an expectation value. Further, but again similar to the example system W, the MoM prediction does again not indicate this discrepancy, even though it is able to predict the system’s escaping behavior.

From a conceptional viewpoint, the CRNs resulting from the two choices of reaction rate parameters differ substantially: Setting $c_{1}=c_{2}=1$ leads to a system with fat-tail steady-state distribution, while $c_{1}=0$ and $c_{2}=1$ results in a system that does not have a steady-state distribution at all. We anticipate that $c_{1}=c_{2}$ is in a sense a bifurcation point that divides parameter combinations with finite escape time ($c_{1}<c_{2}$) from CRNs that converge to an equilibrium distribution. For any parameter choice $c_{1}=c_{2}$, distribution (8) describes the true CME solution of system (11). However, a deeper look into the steady-state solutions of the respective system is needed to make assumptions about the shape of the steady-state distributions arising from $c_{1}>c_{2}$.

### 2.4 Expectation-based methods in fat-tail applications

As indicated earlier, we are interested to test how the MoM behaves when applied to the two chemical Systems P and W. Using Equation (5) and (6), we derive the MoM ODEs for both simulated systems. For System P, this leads to


(18) $$\begin{array}{cc} \frac{d{\tilde{E}}_{x}(t)}{dt} & =10-{\tilde{E}}_{x}(t) \end{array}$$

(19) $$\frac{d{\tilde{V}}_{x}(t)}{dt}=10-2{\tilde{V}}_{x}(t)+{\tilde{E}}_{x}(t).$$

Similarly, the MoM ODEs for System W are derived as


(20) $$\begin{array}{c} \frac{d{\tilde{E}}_{x}(t)}{dt}=1 \end{array}$$

(21) $$\frac{d{\tilde{V}}_{x}(t)}{dt}=1+2\left({\tilde{E}}_{x}(t)({\tilde{E}}_{x}(t)-1)+{\tilde{V}}_{x}(t)\right).$$

The just-presented equations are complemented with the deterministic initial conditions ${\tilde{E}}_{x}(t=0)=1$ and ${\tilde{V}}_{x}(t=0)=0$, which correspond to the moments of the initial distribution P(x=1,t=0)=1, to create solvable initial value problems.

**Figure 6.**
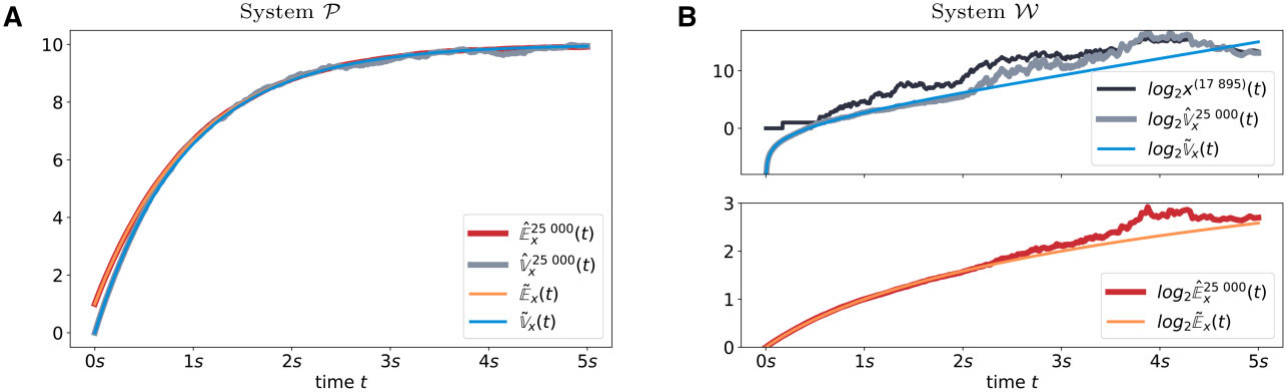
MoM results are qualitatively similar for both models, while sample-based estimators do not agree with them for System W, thereby indicating potentially non-existing moments. Comparison of time-dependent moment estimation for both simulated models. On the one hand, sample-based estimators for $E_{x}(t)$ and $V_{x}(t)$ are depicted in red and light gray, respectively. MoM predictions, on the other hand, are visualized in orange and blue. As for Fig. 4, trajectory $x^{\left(17\;895\right)}(t)$ is plotted in dark gray to show how closely the sample-based variance estimation ${\hat{V}}_{x}^{25\;000}(t)$ is related to this trajectory.

Figure 6 compares the time-dependent solution of the aforementioned equations to the bias-free moment estimators (14) and (15). Both the MoM solution and the sample-based estimators are evaluated over a grid of time points that are logarithmically equidistantly spaced so that time points are more densely distributed for smaller times. For the reference System P depicted in Fig. 6A, the MoM prediction perfectly agrees with the sample-based estimators for the entire simulation time. Hence, it can be assumed that both estimation techniques approximate the system’s characteristics properly.

A qualitatively very different picture is painted for System W (Fig. 6B). Initially, the two estimation techniques agree for both moments. However, after approximately 1 s, the sample-based estimation visually differs from the smooth MoM solution, especially for the variance. It is remarkable, that the potentially still existent moments are obviously increasingly difficult to estimate statistically. The quality of the sample-based estimation continues to decrease beyond the point, where System W no longer possesses statistical moments.

The second observation in Fig. 6 is less obvious at first but similarly essential: Even while crossing the point where the system’s statistical moments are no longer defined, the MoM trajectory is unaffected in nature. In other words, the MoM is unable to indicate the unpredictability of the underlying process.

Similar to Fig. 4, we plotted the time course of sample trajectory $x^{\left(17\;895\right)}(t)$. Besides the order of magnitude of the number of simulated molecules, it is remarkable how similar the variance estimation ${\hat{V}}_{x}^{25\;000}(t)$ resembles this trajectory, especially after 2 s of simulated time. Hence, this single sample dominates the overall estimation process, which inherently questions the usability of statistical moments in such a context.

In summary, results show that the application of the MoM to CRNs that show wildly stochastic behavior provides smooth moment estimates that are similar to moment courses of systems with well-defined moments. Hence, MoM moment trajectories are not indicative of fat-tailedness. In contrast, the consistency of moment estimates from SSA trajectories is lost over time, and single SSA trajectories with large molecule numbers dominate the estimates of both moments. A visual indicator of this is the increasing ruggedness of the estimated moment trajectories over time.

### 2.5 Wildly stochastic behavior results in fat-tailedness of simulation run-times

Our results outlined the difficulties of identifying and predicting wildly stochastic CRNs. The two challenges clearly are related, as the identification of fat-tailed CME solutions is the basis for a subsequent adequate choice of approximation methods. In this chapter, we would like to present a diagnosis approach while simultaneously discussing another interesting aspect of wildly stochastic behavior in the context of CRNs.

To this end, we revisit the SSA used to draw sample trajectories from the CME solution. Starting from the initial configuration P(x,t=0), the algorithm samples the index *j* of the next reaction and a waiting time τ. The waiting time is drawn from an exponential distribution $\text{Exp}(\lambda =\sum_{j=1}^{J}a_{j}(x(t)))$. This distribution has expectation value $\lambda^{-1}$.

**Figure 7.**
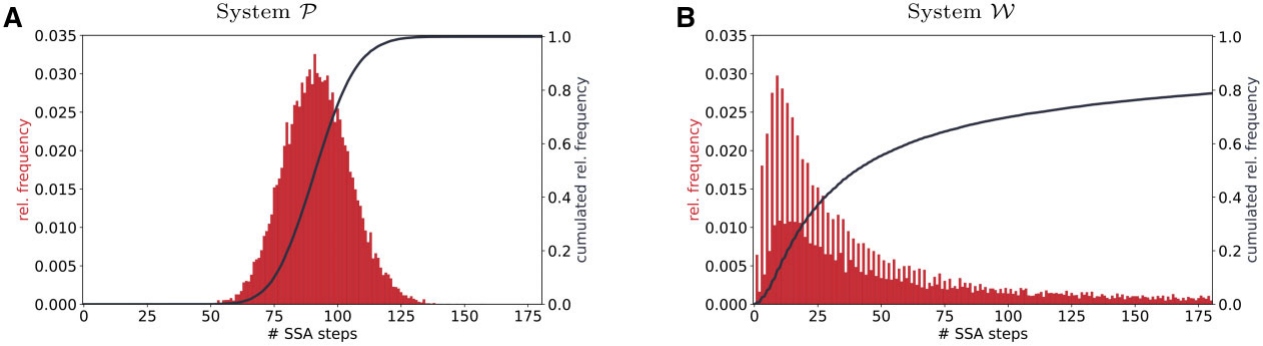
Fat-tailedness of the CME solution translates to the distribution of run times. The histograms describe the number of reactions needed to simulate 5 s of simulated time. Both systems are simulated 25 000 times. The relative frequency is depicted as a red histogram while its cumulative distribution is visualized as a black line. The two quantities are plotted on different scales. (A) Results for Systems P, (B) results for System W.

The number of needed SSA steps to reach 5 s of simulated time is displayed as a histogram over all 25 000 sampled SSA trajectories for both systems in Fig. 7. For System P (Fig. 7A), the histogram resembles a discretized normal distribution with a mean of approximately 90 SSA steps per simulation. Moreover, the probability of one simulation trajectory with less than 40 or more than 140 steps within 5 s of simulated time is approximately zero. In contrast to this, the histogram depicted in Fig. 7B is remarkably different in several aspects. Firstly, it is overall more likely that System W needs an odd number of steps to surpass 5 s of simulated time. While this may sound surprising at first, it can be related to the very small sum of propensities associated with configuration x=1 that translates to a long resting time. In fact, Fig. 2B shows that over 40% of all SSA trajectories finish in this configuration. No matter how their journey through configuration space went until then, they are bound to need an odd number of reactions to surpass the point of 5 s.

The second aspect worth discussing is the long histogram tail in Fig. 7B. It originates from the two quadratic propensities that lead to vanishing expected time increments for large numbers of simulated molecules. Different realizations of System W, therefore, exhibit completely different behaviors: While some trajectories only take one SSA step to reach 5 s of simulated time, other trajectories escape the regime of small molecule numbers (and therefore propensities). Once a certain number of molecules is reached, the system performs an incredible number of reactions to progress only slightly in time. The probability of such an escape from small molecule numbers can be deduced from the black cumulative distribution curve in Fig. 7B. It indicates that more than 20% of all SSA samples are not finished after 175 reactions.

The number of performed SSA steps is directly correlated with the simulation run-time. A scientist attempting to draw samples from a fat-tailed CME solution will therefore experience many very fast simulations at first. However, the overall run-time is completely dominated by single extreme events. The simulation of trajectory $x^{\left(17\;895\right)}(t)$, for instance, took several days, while most of the 25 000 samples were completed within a fraction of a second. Besides the unpleasant observation of such extreme samples, the distribution of run-times like the one depicted in Fig. 7 can already unveil the fat tails of the CME solution itself.

## 3 Discussion and conclusion

The CME is widely recognized for accurately describing the behavior of CRNs of various different types and characteristics. Due to this flexibility, it is in principle not surprising that the CME solution of particular systems does not possess any statistical moments. However, it still is counter-intuitive that those systems can be derived from simple mass action kinetics of second-order reactions.

In this work, we first derived a CRN that provably converges to a fat-tail power-law distribution. After slightly altering the system to increase interpretability, we numerically validated our theoretical claims. On this basis, we presented evidence that expectation-based CME approximation methods are prone to fail when applied to systems that converge to fat-tail distributions. Moreover, the MoM does not indicate its failure by any means. In our eyes, it is, therefore, crucial to examine each CRN thoroughly before choosing an adequate approximation technique for the CME. To this end, we propose to employ a sample of SSA simulations. The corresponding implementation is simple, yet, SSA trajectories represent a true sample of the underlying CME that can be used in different ways for a moment diagnostics. As demonstrated, applying moment estimators to the sampled trajectories and observing their convergence can indicate whether or not statistical moments of the CME solution exist and how easily they can be estimated. Another interesting aspect of the SSA is that its run-time scales linearly with the number of recorded reactions. In the context of our simulations, extreme events that effectively prevent the CME solution from having statistical moments correspond to simulations where the system escapes the slow dynamics of a few molecules, thereby further increasing the reaction propensities quadratically. As indicated earlier, this vast amount of reactions manifests itself through surprisingly large simulation run-times. Effectively, the fat tail of the CME solution is therefore transferred to the distribution of simulation run-times, which is a quantity most simulation researchers attentively monitor and which therefore serves as another criterion to assess the system’s expectability.

Beyond the methods that we have used in the article, other diagnostic methods to characterize the CME solution’s moments are conceivable. For example, traditional outlier testing like Chauvenet’s or Peirce’s criterion and Grubbs’s test could be applied to SSA simulations to indicate extreme results.

## Data Availability

All code is available via https://fairdomhub.org/models/827 and can be executed with any common ‘Python 3’ distribution. Necessary packages are publicly available.
